# Tegument Protein pUL47 Is Important but Not Essential for Horizontal Transmission of Vaccinal Strain SB-1 of Gallid Alphaherpesvirus 3

**DOI:** 10.3390/v17030431

**Published:** 2025-03-18

**Authors:** Motoyuki Esaki, Mélanie Chollot, Sylvie Rémy, Katia Courvoisier-Guyader, Zoltan Penzes, David Pasdeloup, Caroline Denesvre

**Affiliations:** 1 Ceva Santé Animale, Ceva-Japan, Yokohama, Kanagawa 230-0045, Japan; moto.esaki@ceva.com; 2 Equipe Biologie des Virus Aviaires, UMR1282 ISP, INRAE, 37380 Nouzilly, France; melanie.chollot@inrae.fr (M.C.); katia.guyader@inrae.fr (K.C.-G.); david.pasdeloup@inrae.fr (D.P.); 3 Ceva Santé Animale, Ceva-Phylaxia, 1107 Budapest, Hungary; zoltan.penzes@ceva.com

**Keywords:** *Orthoherpesviridae*, avian viruses, GaHAV-3 SB-1, vaccine, chicken, UL47, latency, feathers, transmission

## Abstract

The gallid alphaherpesvirus 3 (GaAHV3) SB-1, a *Mardivirus* used as a vaccine against Marek’s disease, has been proposed as an interesting viral vector for poultry vaccination. However, SB-1 is highly transmissible between chickens, a feature that may be a limitation for the use of live recombinant vaccines. We have previously shown that UL47 is essential for horizontal transmission of the pathogenic Marek’s disease virus between chickens, but it is completely dispensable for replication and pathogenesis. In contrast, the role of UL47 in the biology of SB-1 remains unknown. To study that, we generated an SB-1 mutant lacking UL47 (∆47) from a commercial SB-1 isolate. This mutant replicated and spread like the WT in primary fibroblasts, indicating no growth defects in cell culture. In vivo, chickens inoculated with ∆47 had significantly reduced viral loads in the blood and the spleen, and transport to the skin was delayed compared to WT inoculated chickens. Strikingly, the ∆47 mutant was present in 66% of contact birds. As expected, 100% of contact birds were positive for the WT. In conclusion, our findings reveal that UL47 facilitates GaAHV3 SB-1 replication in vivo, which is important for latency establishment but is not essential for horizontal transmission, unlike for MDV.

## 1. Introduction

Gallid alphaherpesvirus 3 (GaAHV3) is a non-pathogenic virus in chickens identified for the first time in 1978 and now classified within the *Orthoherpesvirus family*, the *Alphaherpesvirinae subfamily*, genus *Mardivirus* [1]. Since the early 1980s, GaAHV3 has been used in some countries as a live vaccine to protect against Marek’s disease (MD), caused by the highly oncogenic Marek’s disease virus (MDV, GaAHV2 strain, gallid alphaherpesvirus 2) [2]. GaAHV3 is often commercialized as a vaccine in combination with the herpesvirus of turkey (HVT; meleagrid alphaherpesvirus 1), another non-pathogenic *Mardivirus* for chickens [2]. GaAHV3, MDV, and HVT share several genetic, serologic, and biological properties [3]. Indeed, GaAHV3’s genome shares approximately 61% sequence identity with that of MDV [4]. Regarding biology, all three virus species establish latency in T-cells, allowing for lifelong persistence in chickens, and replicate in the feather follicle epithelium (FFE) [1,5,6,7,8,9].

Several viral vectored vaccines have been developed in poultry over about 40 years [10]. Today, HVT is one of the most widely used vaccine platforms [10]. Various foreign antigens have been introduced into its genome to protect against other major viral diseases of poultry, such as infectious bursal disease (IBD), Newcastle disease (ND), or avian influenza (AI) [10,11,12,13,14]. Recombinant HVT vaccines (e.g., HVT-ND or HVT-IBD) are very effective in protecting against each disease when injected alone. However, when several recombinant HVT vaccines are administered together, interference has been reported, resulting in lower protection than the recombinant HVT administered alone (reviewed in [10]). To overcome this limitation, double and triple insert HVT vaccines have been developed in the last 10 years [15,16,17]. An additional solution to cancel out this limitation would be to associate a recombinant HVT with a recombinant derived from another virus species. The GaAHV3 SB-1 strain (hereafter referred to as SB-1) presents itself as a promising candidate, having previously been identified as a potential recombinant viral vector vaccine for poultry [18]. Such an alternative is also supported by the fact that SB-1 has been used for a long time in association with HVT to protect efficiently against MD [2,19,20], indicating the compatibility and benefit of associating the two live viruses. However, a drawback of using SB-1 as a vector could be its high potential of transmission. Indeed, SB-1 virus is highly shed in poultry dust and horizontally transmissible between chickens. This has been documented experimentally either with SB-1 alone [1] or with the SB-1 vaccine in combination with HVT [19,21]. In addition, GaAHV3 viruses have been detected in the field in samples from non-SB1-vaccinated chickens, indicating the silent circulation of such viruses among chickens, whether vaccinated or not, and highlighting the great potential for the spread of GaAHV3 [6,22,23,24].

Therefore, identifying SB-1 genes essential for horizontal transmission would be of great interest to generate new backbone vectors and to avoid uncontrolled transmission of recombinant vaccines.

Recently, for MDV, we identified UL47, a 2.4 kbp long gene, encoding the tegument protein pUL47 (VP13/14), which is dispensable for replication in cell culture and in chickens in peripheral blood mononuclear cells (PBMCs) and FFE but essential for horizontal transmission of MDV between chickens [25]. The role of UL47 in SB-1 replication has not been reported yet.

In this study, we investigated the role of UL47 in SB-1 replication in cell culture and in vivo, particularly for horizontal transmission in chickens. Our data reveal that in the absence of UL47, SB-1 replication in vitro remained unaffected, while replication in vivo was moderately attenuated. Furthermore, UL47 is not essential for horizontal transmission of SB-1 between chickens, in contrast to MDV.

## 2. Methods

### 2.1. Ethics Statement

The in vivo experiment was carried out according to the guidance and regulation of the French Ministry of Higher Education, Research and Innovation (MESRI), with appropriate staff, good animal practices, and project authorizations (Protocol Number APAFIS#31071-2021041615377018 v3). As part of this process, the experimental protocol was thoroughly examined and approved by the appropriate local ethics committee, CREEA VdL (“Comité d’Ethique pour l’Expérimentation Animale Val de Loire”).

### 2.2. pUL47 Sequence Alignment

Sequence alignment of SB-1 pUL47 with seven other alphaherpesviruses was performed using the MUSCLE 3.8.425 program and the Clustal algorithm (https://www.ebi.ac.uk/jdispatcher/msa/muscle?stype=protein, accessed on 8 August 2024). These alphaherpesviruses are MDV, HVT, the infectious laryngotracheitis virus of chicken or gallid alphaherpesvirus 1 (GaAHV1), bovine alphaherpesvirus 1 (BoAHV1), human simplex virus 1 (HSV1/human alphaherpesvirus 1), varicella-zoster virus (VZV/human alphaherpesvirus 3), and pseudorabies virus (PRV/suid alphaherpesvirus 1).

### 2.3. Cells

Chicken embryonic fibroblasts (CEFs) were prepared from 10- to 11-day-old embryonated eggs of specific pathogen free (SPF) chickens according to standard procedures [26]. In brief, CEFs were cultivated in Leibowitz–McCoy (LM, 1:1) medium with 4% bovine calf serum at 37 °C.

Chicken embryonic skin cells (CESCs) were prepared from 12-day-old embryonated eggs of SPF White Leghorn chickens (INRAE), as described previously [27]. In brief, CESCs were cultivated in William’s modified E medium with 2% chicken serum and 3% fetal calf serum (FCS) at 41 °C.

### 2.4. Generation of SB-1 Mutant and Virus Propagation

SB-1 virus lacking the UL47 gene (∆47) was generated using the Ceva SB-1 commercial vaccine strain. First, the pSB1-deltaUL47 plasmid was constructed. It contains the UL46 and the UL48 genes of SB-1 but lacks the entire UL47 gene (sequence on demand). Next, the deletion was performed by co-transfecting CEFs with SB-1 viral DNA prepared as described by Morgan [28] and the pSB1-deltaUL47 plasmid through electroporation using Nucleofector^®^ II (Lonza, Switzerland). Transfected CEFs were plated on 96-well plates and incubated at 37 °C for 5–7 days until plaques were observed. Cells were harvested through trypsinization from wells that contained plaques. Half of the cells were used to pass the virus to fresh CEFs, and the other half was used to extract DNA. DNA was extracted by incubating the cells in the lysis buffer (20 mM Tris-HCl, pH 8.0, 100 mM NaCl, 5 mM EDTA, 0.1% SDS, and 0.1 mg/mL Proteinase K) for 10 min at 60 °C and 2 min at 98 °C. The extracted DNA was used in PCR to detect UL47 deletion using two sets of primers (SB1UL48-F/SB1UL46-R; SB1UL47-1-F/SB1UL47-1-R) (Table 1). Clones containing the UL47 deletion were cultivated until the next screening, and the other clones were discarded. This purification procedure was repeated until no parental virus was detected through PCR. Two clones with deletion of UL47 (named clones 1 and 2) were pooled to generate the SB-1 virus with deletion of UL47 (∆47 mutant). The mutation was subsequently confirmed through Sanger sequencing. The ∆47 mutant virus was next amplified 3 times on CEFs. The WT SB-1 (Ceva strain) was at passage +30, a passage higher than the commercial vaccine to be at the same level as the deletion mutant. Both viruses (WT and ∆47) were titrated for animal experiments. The absence of UL47 was checked again from viral DNA prepared from infected CESCs, as described earlier [29,30], through PCR with UL51 and UL47 primers (SB1UL47-2-F/ SB1UL47-2-R) (Table 1).

### 2.5. Multi-Step Growth Kinetics

To start, 0.6 million CESCs were infected with 100 PFU in 6-well plates of the respective SB-1 viruses (WT and ∆47) and cultured over 5 days. Every day, the content of one well per virus was harvested for qPCR and stored at −80 °C. Next, DNA from all samples was extracted using the NucleoSpin Tissue DNA mini kit (#740952, Macherey-Nagel, Hoerdt, France) according to the manufacturer’s instructions, and SB-1 replication was assessed based on virus quantification through real-time PCR (see below).

### 2.6. Plaque Size Assays

The replication properties of the ∆47 mutant were assessed using plaque size assays, as described before for MDV [31]. CESCs, seeded in 6-well plates, were infected with 100 pfu of the indicated SB-1 viruses (WT and ∆47). At 4 dpi, cells were fixed with 4% paraformaldehyde for 20 min, permeabilized with 0.1% triton X-100, and blocked with 1% serum albumin bovine. The plaques were next stained using a chicken serum collected on a SB-1-vaccinated chicken diluted 1:1000 for 1 h at room temperature and next with a goat anti-chicken Alexa Fluor 488 antibody (Invitrogen, Illkirch-Graffenstaden, France). Images of 50 randomly selected plaques per condition were obtained on an Axiovert 200M inverted microscope (Zeiss, Göttingen, Germany) with a 5x fluar long-distance objective. The area of imaged plaques was determined using Axiovision LE64 software (release 4.9.1, Zeiss).

### 2.7. Animal Experiment

Thirty-seven one-day-old SPF White Leghorn chickens (B13/B13 haplotype) were obtained from the INRAE animal facility and randomly distributed into two groups (WT, *n* = 19; ∆47, *n* = 18). In each group, half of the chicks were vaccinated subcutaneously with 4000 pfu of SB-1, either WT (*n* = 10) or ∆47 (*n* = 9). This dose was chosen to be similar to the appropriate dose for the commercial SB-1 vaccine. Of note, the virus inoculums were not back-titrated. Nine chicks per group were non-vaccinated and serve as contact chickens (contacts). The two groups (WT and ∆47) were housed separately in two independent isolators for nine weeks. Whole blood and 2–3 growing feathers were collected from all inoculated chickens at 14, 28, 42 and 56 dpi. Whole blood was collected from all contact chickens at 21, 28, 35, 42, and 56 dpi. One chicken from the WT group died suddenly at 46 dpi (no macroscopic lesions at necropsy) and did not contribute data thereafter. All birds were humanely euthanized at the end of the experiment (63 dpi) and necropsied. At necropsy, spleens were harvested from each chicken. In addition, dust samples were collected from air filters in each isolator at 21, 28, 35, 42, and 56 dpi and stored at −20 °C before analysis. The dust collected on day 21 represents the total dust accumulated since the beginning of the experiment. The dust collected on day 28 represents the dust accumulated between days 21 and 28, and so on, until the end of the experiment. Peripheral blood monocyte cells (PBMCs) from the whole blood and mononuclear cells from the spleen (named herein splenocytes) of each chicken were purified on a Ficoll-based lymphocyte separation medium (CMSMSL01-01, Eurobio Scientific, Les Ulis, France), as previously reported [32].

### 2.8. DNA Extraction from PBMCs, Splenocytes, Feather Samples, and Dust

DNA was extracted from PBMCs, growing feather tips (proximal ends containing feather pulp and epithelium) and splenocytes using the QIAamp DNA mini kit (Qiagen, Germantown, MD, USA), as described previously [32,33] and according to the manufacturer’s instruction. For dust samples, DNA was extracted from 10–20 mg using the QIAamp DNA mini kit or the NucleoSpin Tissue DNA mini kit (#740952, Macherey-Nagel).

### 2.9. Virus Quantification Through Real-Time PCR in PBMCs, Splenocytes, Feather Samples, and Dust

SB-1 genome copies were quantified through real-time PCR using TaqMan technology and reported relative to one million cells. SB-1 (DNA Pol serotype 2) and chicken iNos primers and FAM-BHQ1 tagged probes (DNA Pol serotype 2 and iNos) were previously described [7,34] (see Table 1) and purchased from Eurogentec. For the qPCR SB-1 standard, a synthetic gene of 300 bp from SB-1 DNA pol was generated and cloned in pMA-T by Thermo Fisher Scientific (sequence available on demand). With the SB-1 standard, the threshold of detection was 16 copies. The genome copy number was normalized to one million cells using a standard curve for iNos generated with a standard plasmid (sequence available on demand). All qPCRs (except for dust, see below) were performed independently in triplicates, for SB-1 and for iNos, with 250 ng DNA, 10 pmol of each gene-specific primer, and 5 pmol of the gene-specific probe in a 20 μL volume on a CFX96™ Real Time C1000 Touch™ Thermal Cycler (BioRad, Marnes-la-Coquette, France). The results were analyzed using CFX Manager software (version 3.1) (BioRad). The table with all individual viral loads is available on demand.

For dust, qPCR was performed in triplicates with 100 ng of DNA using SB-1 primers and probe only, and the absolute number of genome copies was calculated using the SB-1 standard.

### 2.10. Viral PCR Analyses

For splenocytes, PCRs were performed on 100 ng of DNA in a volume of 25 µL using the GoTaq DNA polymerase (Promega, Madison, WI, USA) according to the manufacturer’s instructions, with the following program: 3 min of denaturation at 94 °C, annealing for 30 s (54 °C for UL47; 56 °C for UL51), and extension at 72 °C for 30 s, with 35 cycles. A final extension of 5 min was performed at the end of the program. The viral products were deposited on 1% agarose gel.

### 2.11. Statistical Analyses

Statistical analyses were performed using GraphPad Prism (v. 7; GraphPad Software, Inc., La Jolla, CA, USA) or R software (v.3.4.3.), as indicated in the figure legends. Non-parametric tests were used for the data analysis of the in vivo experiment because of small sample sizes (9–10 independent subjects per virus). All common applied statistical tests are indicated in the respective figure legends. For the analysis of variance in the case of mixed models (e.g., two viruses at different dates), a non-parametric ANOVA-like test for non-parametric analysis of longitudinal data in factorial experiments using ranks and adjusted *p*-values for pairwise comparisons was used [35,36]. Data were considered significantly different if *p* ≤ 0.05. GraphPad prism version 7 was used for plots.

## 3. Results

### 3.1. UL47 Is Dispensable for Vaccine SB-1 Replication In Vitro

**Figure 1:**
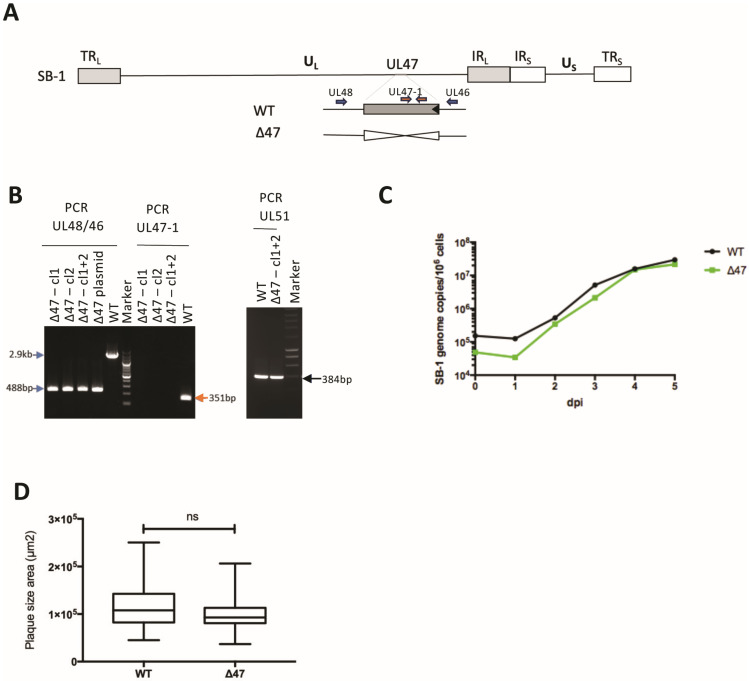
Generation and characterization of ∆47 SB-1. (**A**) Schematic representation of the SB-1 genome with the UL47 deletion, resulting in the Δ47 mutant. The positions of the UL46 and UL48 primers are shown (blue) as well as the UL47-1 primers (orange). (**B**) PCR analysis of indicated viruses cultivated in vitro, with indicated primers to check for UL47 deletion. Two clones (#1 and #2) of ∆47 SB-1 were tested and pooled for all subsequent experiments. (**C**) Multi-step growth kinetics of the WT and Δ47 SB-1 between one and five days post-infection (dpi) in CESCs. This graph is representative of two independent experiments. (**D**) Plaque size assays of indicated viruses 5 days post-infection in CESCs. The box plots depict median plaque diameters with the minimum and maximum values. Statistical analysis (exact *p*-value = 0.0793, Mann–Whitney test) was performed with a sample of 50 plaques per condition. The results of a unique experiment are shown, representative of two independent experiments.

The SB-1 UL47 gene is 2436 bp long and encodes a protein of 811 amino acids (AA). pUL47 SB-1 has 50.3% AA identity with its MDV RB-1B orthologue (808 AA long) using the MUSCLE 3.8.425 program and the Clustal algorithm (https://www.ebi.ac.uk/jdispatcher/msa/muscle?stype=protein, accessed on 8 August 2024). The AA identity is lower with HVT (44.9%) and with alphaherpesviruses from other genera (between 17 and 25%). This low AA identity between pUL47 MDV and pUL47 SB-1 prompted us to question whether the role of pUL47 is conserved between the two *Mardivirus* species. To assess the role of UL47 in GaAHV3 SB-1 replication and transmission, we deleted the UL47 gene from the SB-1 vaccine strain (Figure 1A) through homologous recombination in cell culture. For that, viral SB-1 DNA was first co-transfected in CEFs with the pSB1-deltaUL47 plasmid. Next, DNA was extracted from individual viral plaques and screened for the absence of the UL47 gene through PCR. UL51 was used as a control gene. Deleted clones were expected to show a band at 488 bp with UL48/46 primers, no band with UL47-1 primers, and a 348 bp band with UL51 primers. Two clones (1 and 2) harboring the UL47 deletion were selected and pooled (Figure 1B). The resulting SB-1∆47 mutant virus (named thereafter ∆47) was amplified on CEFs. The mutant was verified through PCR compared to the two initial clones (Figure 1B), as well as through Sanger sequencing of the 488-bp fragment amplified with the UL48/46 primers.

Next, we assessed whether deletion of UL47 affected virus replication in cell culture. Multi-step growth kinetics revealed that ∆47 replicates similarly to the WT SB-1 in CESCs (Figure 1C). Furthermore, no significant difference in spread was observed between the two viruses, ∆47 and WT, in CESCs based on plaque size assays (Figure 1D). Taken together, our data demonstrate that UL47 is dispensable for vaccine SB-1 replication in cell culture.

### 3.2. The Absence of UL47 Reduces SB-1 Viral Load in the PBMCs and the Spleen of Inoculated Chickens

**Figure 2:**
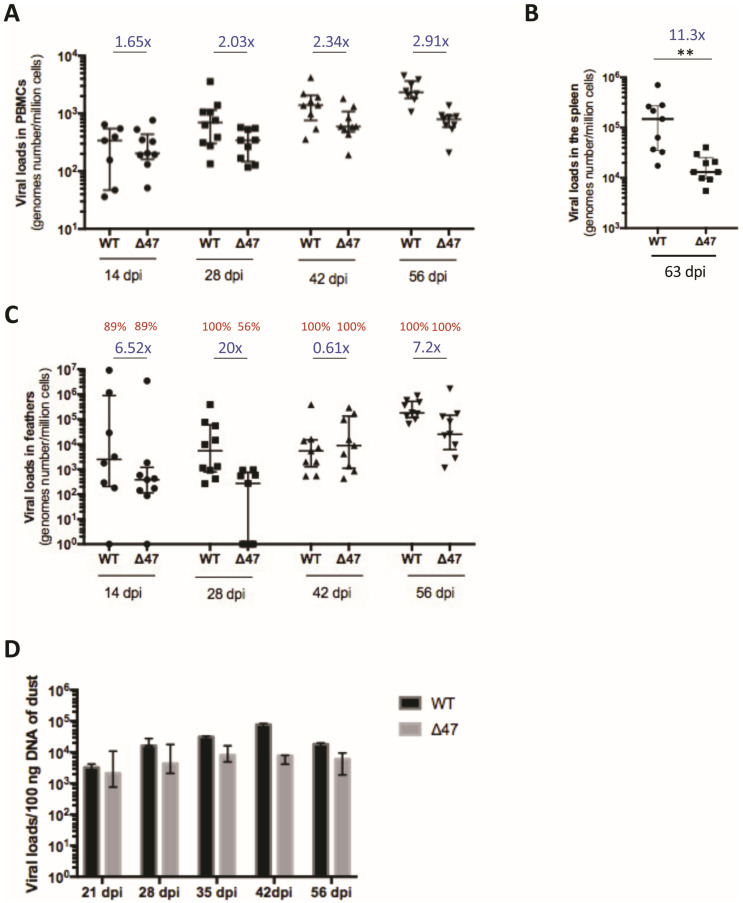
The absence of UL47 moderately reduces SB-1 loads of PMBCs and the spleen of inoculated chickens. (**A**–**C**) SB-1 genomes were quantified through qPCR in PBMCs (**A**), splenocytes (**B**), and feathers (**C**) of chickens inoculated with WT or Δ47 at indicated time points (14, 28, 42, 56, or 63 dpi). Different symbols are used at each time points. Data are shown as dots with the median and the interquartile range. The fold-change between medians (WT/∆47) is shown in blue for each time point. (**A**) In PBMCs, the difference between the two viruses is significant (*p* = 0.0051, permutations mixed model for repeated measures), as well as the interaction between the virus and time (*p* = 0.0001, permutations mixed model for repeated measures). (**B**) The median load in splenocytes was significantly reduced by 11.3-fold in the absence of UL47 (*p* = 0.0012 (** <0.01), Mann–Whitney test, two-tailed). (**C**) In feathers, the difference between the two viruses was not significant (*p* = 0.52, permutations mixed model for repeated measures), nor was the interaction between the virus and time (*p* = 0.85, permutations mixed model for repeated measures). The percentage of birds with a detectable virus genome is shown in red above each group measure (e.g., 56% of the contact chickens of the mutant group had detectable SB-1 genome in feathers at 28 dpi). (**D**) Absolute quantification of SB-1 genome with qPCR on 100 ng of DNA extracted from dust material. The graph results are from 3 independent experiments of extraction and qPCR at each time point (*n* = 3). Data are shown as bars with the median and the interquartile range at indicated time points for both virus groups.

To assess the role of UL47 in vivo, we subcutaneously inoculated one-day-old chickens with 4000 pfu of SB-1, either ∆47 or WT. First, we quantified viral genome copies in peripheral blood mononuclear cells (PBMCs) of the inoculated chickens through absolute qPCR over time, as the virus infects, replicates, and integrates into the chromosomes of lymphocytes [8]. All of the PBMCs samples were SB-1 positive. Nevertheless, the ∆47 genome copy numbers were significantly reduced in PBMCs (between 1.6- and 2.9-fold) from 14 to 56 dpi compared to the WT (Figure 2A). Taken together, our data revealed that the SB-1 genome copies were moderately reduced but significantly reduced in PBMCs in the absence of UL47. This indicates that UL47 is non-essential for SB-1 replication in vivo but involved in the efficiency of PBMC infection, replication, and/or integration.

We also quantified the viral loads in splenocytes purified from the spleens of inoculated birds at the end of the experiment (63 dpi). A significant reduction (11.3-fold) in SB-1 genome copies was detected in the absence of UL47 compared to the WT (Figure 2B). This indicates that the absence of UL47 moderately impairs latency establishment and/or maintenance in the spleen.

### 3.3. The Absence of UL47 Delays Virus Transport to the Skin of Inoculated Chickens but Does Not Compromise Replication and Persistence in Feathers

During the course of infection, SB-1 is transported to feather follicle epithelial (FFE) cells in the skin, from where it replicates and is shed into the environment [7,37]. To assess virus transport and replication in the skin, viral genome copies were measured through qPCR in feathers collected from inoculated birds over time, from 14 dpi to 56 dpi. The ∆47 genome copy numbers in feathers showed variations compared to the WT (20-fold at 28 dpi, 0.61-fold at 42 dpi, and around 7-fold at 14 and 56 dpi). The kinetics showed that the transport to the feathers was delayed in the absence of UL47 when compared to the WT, as, at 28 dpi, 56% of birds inoculated with ∆47 had a detectable genome in their feathers, whereas 100% were already positive with the WT (Figure 2C). At 42 and 56 dpi, 100% of the feather samples were positive for both viruses, with no significant difference in virus loads, although this was at the limit of the fixed significance threshold (*p* = 0.52). Taken together, our results revealed that UL47 plays a role in the dynamics of reaching the feathers but not in replication or persistence in this tissue.

In order to examine the shedding of both viruses into the environment, dust samples were collected from the two isolation units at five time points post-infection, and total DNA was extracted three times independently. For both viruses, the viral loads were quantified in dust between 21 and 56 dpi. Viral loads varied between 10^3^ and 8 × 10^4^ per 100 ng of DNA, with some variations between independent repeats. Such variations could be due either to the fact that dust is not a homogenous material and/or variable PCR inhibitors (a well-known characteristic of such material). Graphically, no or low differences of viral loads in dust were visible between the two viruses between 21 and 35 dpi, the times at which transmission may occur (Figure 2D). At 42 dpi, the viral loads appeared slightly increased in SB-1 compared to ∆47. Statistical analysis was not feasible herein due to the low number of samples. Together, our results indicate that viral genomes were present in dust for both viruses at all times, with comparable or small differences in amounts.

### 3.4. Tegument Protein pUL47 Is Important but Not Essential for Horizontal Transmission Between Chickens

**Figure 3:**
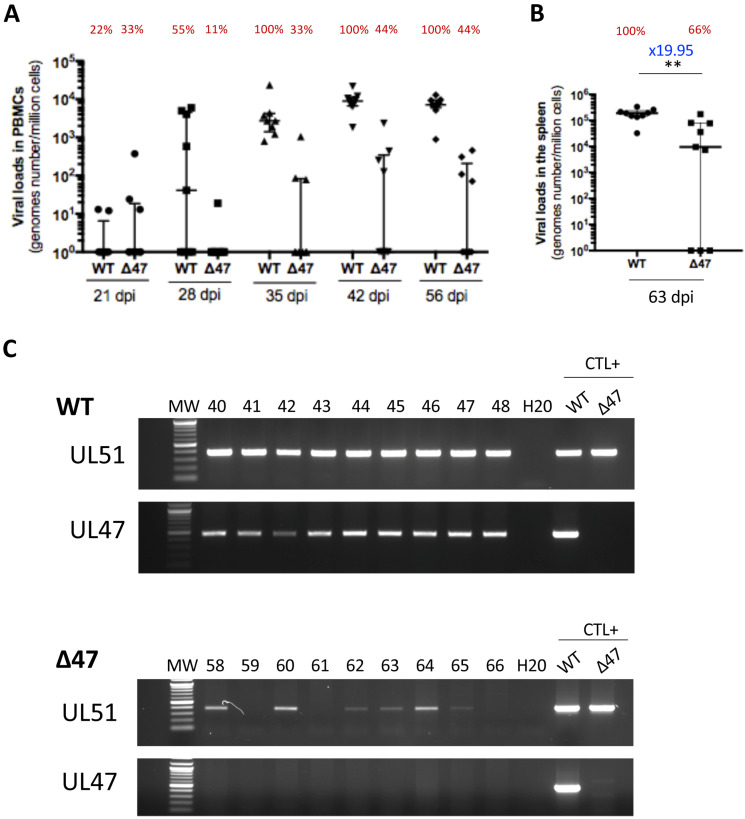
The absence of UL47 reduces SB-1 transmission to contacts but does not abolish it. (**A**–**C**) SB-1 genomes were quantified through qPCR in PBMCs (**A**) and splenocytes (**B**) of contact chickens from the WT or Δ47 group at indicated time points (14, 28, 42, 56, or 63 dpi). Data are shown as dots with the median and the interquartile range. The percentage of birds with a detectable virus genome is shown in red above each group measure (e.g., 22% of the contact chickens of the WT group had a detectable SB-1 genome in PBMCs at 21 dpi). (**A**) Dynamic of SB-1 load in PBMCs over time. The difference between the two viruses is significant (*p* = 0.0001, permutations mixed model for repeated measures), as well as the interaction between the virus and time (*p* = 0.0001, permutations mixed model for repeated measures). (**B**) SB-1 loads in the spleen of contact chickens at the end of the experiment (63 dpi). The median load was significantly reduced by 11.3-fold in the absence of UL47 (*p* = 0.0018 (** <0.01), Mann–Whitney test, two-tailed). (**C**) PCR of DNA extracted from splenocytes using UL47-2 and UL51 primers. Viral DNA extracted from CESCs infected with SB-1 or ∆47 was used as the positive control. The UL47 PCR was negative in all spleens from the ∆47 group.

To investigate the role of UL47 in SB-1 transmission between chickens, inoculated chickens were raised with contact chickens of matched age from the beginning of the experiment. The contact chickens were monitored for virus loads through qPCR in PBMCs over time. The SB-1 genome was detectable in the PBMCs of all contact birds of the WT group from 35 dpi, with viral levels even higher than those in inoculated birds (Figure 3A). This result suggests that the WT is more infectious when chickens are infected through the respiratory route rather than the subcutaneous route. The WT genome was persistent in PBMCs until the end of the experiment, as expected, with most viral loads between 10^3^ and 10^4^ genomes/million cells. The ∆47 genome was also detectable in the PBMCs at 35 dpi, but only in 33% of contact birds. In this ∆47 group, at later time points (42 and 56 dpi), the virus was present in PBMCs of 44% of contact birds at lower levels than in the WT group, with most viral loads between 10^2^ and 10^3^ genomes/million cells. In the ∆47 group, four birds (44%) never showed any positivity in PBMCs during the experiment. Transmission to contact birds was also confirmed by the detection of the virus genome in the splenocytes of 66% of ∆47 and 100% of the WT (Figure 3B). Surprisingly, one ∆47 bird (#65) showed a virus genome detectable in the spleen (above 10^2^ genomes/million cells on two independent DNA extractions), whereas no virus genome was detected in the PBMCs at all time points. Such a result suggests that we may have missed the time at which the virus was replicating in the PBMCs and that the virus was persisting at too low of a level in PBMCs to be subsequently detected. Finally, the virus loads’ median in the spleen was significantly lower (19.95-fold) in the absence of UL47 compared to the WT (Figure 3B). This suggests that in contacts, such as in inoculated birds, the absence of UL47 impairs the establishment and/or maintenance of latency in the spleen.

In order to rule out that virus transmission in the ∆47 group was due to a contamination of the ∆47 virus stock by the WT virus, PCRs of DNA extracted from splenocytes of all contact birds (∆47 and SB-1) were performed on UL47 and UL51 genes. As expected, PCRs using UL51 primers were positive for all infected chickens, which is 100% of the WT and 66% of the ∆47 (Figure 3C). The birds for which no viral genome was detectable through qPCR were also negative based on the PCR with UL51. PCRs using UL47-2 primers were positive for all contact chickens from the WT group and none from the ∆47 one. These data validate that the virus transmitted in the ∆UL47 group between inoculated chickens and contacts did not include some contaminating WT virus. Overall, these data indicate that SB-1 is transmissible between chickens in the absence of UL47 but with a reduced yield compared to the WT. In addition, in the absence of UL47, the viral loads in the blood and spleen were reduced in contact birds, as in the inoculated birds.

## 4. Discussion

UL47 is a conserved gene among the *Alphaherpesvirinae* subfamily encoding pUL47, a tegument protein with limited AA identity in most regions. Several roles have been ascribed to pUL47 in vivo, such as skin tropism for varicella-zoster virus (VZV ORF11) [38], virulence for GaAHV1 (gallid alphaherpesvirus type 1) and BoAHV1 (bovine alphaherpesvirus type 1) [39,40], and virus transmission between natural hosts for MDV [25]. Although not all functions have been studied for all virus species, when data are available, it is interesting to note that some functions are not conserved between species. For example, for MDV, pUL47 is not important for skin tropism and virulence, as assessed based on lymphomagenesis [25]. In this study, we investigated the role of UL47 in GaAHV3 SB-1 in vitro and in vivo. First, we generated SB-1 deletion of UL47 and characterized this ∆UL47 mutant virus in cell culture. Our data showed that SB-1 replicates and spreads efficiently in CESCs in the absence of UL47, indicating that UL47 is dispensable for in vitro replication under the conditions tested, as previously observed for the pathogenic RB-1B MDV [25], for VZV [38], and for GaAHV1 [39]. In this respect, SB-1 differs from Pseudorabies virus (PRV) and BoAHV1, for which deletion of UL47 impaired in vitro replication [40,41].

We next investigated the role of UL47 in SB-1 replication, latency, skin delivery, shedding, and horizontal transmission in its natural host, the chicken. Our results showed that ∆47 replicated in PBMCs of inoculated chickens, as previously observed for MDV [25], although the loads were slightly reduced compared to the WT, a feature that was not observed with MDV. This feature was also observed in infected contact chickens. Hypotheses to explain this result could be that SB-1 lacking UL47 attracts fewer lymphocytes at the site of entry (the dermis in inoculated chickens or the respiratory tract in contact chickens) and/or is attenuated in its replication in lymphocytes.

To examine latency and genome maintenance in lymphocytes, we measured viral loads in the spleen at the end of the experiment. Indeed, virus genome in the spleen at late time points is predominantly in a latent state, although reactivation events cannot be entirely ruled out. The SB-1 load in splenocytes was significantly reduced in the absence of UL47 in inoculated chickens and in infected contacts. This result suggests that UL47 SB-1 is directly or indirectly involved in latency establishment and/or genome maintenance in the spleen. Note that such an observation is not available for MDV for comparison. Considering the known in vitro functions of pUL47, such as virion morphogenesis for PRV and HSV1 [41,42] and RNA binding of virus-specific RNA transcripts for HSV1 [43] and BoAHV1 [44], the reduction in latency observed in the absence of UL47 in the spleen appears to be related to lower viral loads in PBMCs rather than a true reduction in the integration process itself and/or genome maintenance.

Next, we assessed the levels of SB-1 loads in the feathers. Our data demonstrate that UL47 is not necessary for skin tropism, as has been shown for MDV [25], although the delivery of SB-1 to the skin was delayed in the absence of UL47 in inoculated chickens. Because latently infected cells are believed to transport *Mardivirus* to the skin [45], we can speculate that in the absence of UL47, such a delay may be a consequence of the lower levels of SB-1 in blood and spleen lymphocytes.

Finally, we evaluated whether UL47 is involved in SB-1 horizontal transmission between hosts. Contrary to what was observed for MDV [25], the transmission between inoculated and contact chickens was reduced but not abolished. For SB-1∆47, viral genomes were present in dust and at comparable levels to the WT, indicating that virus shedding was not severely impaired in the absence of UL47. In addition, the partial transmission observed for SB-1∆47 cannot be explained by very high loads in feathers and dust because virus loads in these materials were globally lower for SB-1 than for MDV [25]. Therefore, the difference in transmission observed for MDV and SB-1 seems more related to the nature of the shed virions than any alteration in shedding efficiency. Indeed, our findings demonstrate that SB-1 particles lacking UL47 shed in dust are still infectious through inhalation in contrast to MDV∆47 particles. To explain this difference, one hypothesis is that the role of UL47 in transmission is not absolute for SB1 as it is for MDV. For SB-1, this feature may be related to another factor or viral cofactor that partially compensates for the absence of UL47 or is less dependent on UL47 for its function.

Importantly, because the SB-1∆47 mutant exhibits reduced replication in vaccinated chickens while remaining transmissible to contact chickens, it does not appear to be a suitable candidate for a recombinant vaccine vector.

In summary, our study provides evidence that UL47 is not essential for viral replication of GaAHV3 strain SB-1 in vitro or in vivo. Importantly, the SB-1 mutant lacking UL47 is attenuated in horizontal transmission between chickens but not completely impaired like MDV. These findings highlight that UL47 may serve distinct roles and functions across different virus species, including closely related viruses like GaAVH3 and GaAHV2.

## Figures and Tables

**Table 1:** Primers and probes used in this study.

Target Gene		Sequence (5′ to 3′)	Amplicon Size
SB1UL48-F2	for	CTGCTGCTCGCACAATGTAA	2894 kbp with UL47 488 bp wo UL47
SB1UL46-R2	rev	GCTAGCGCAAGCATATCG	
SB1UL47-F1	for	GAAAGTATTGCCCCGGGTAT	351 bp
SB1UL47-R1	rev	CTCATTTAATCGCGACAGCA	
SB1 qPCR	probe	FAM-CCCGGGTCGCCTCATCTGGA-TAMRA	67 bp
	for	AGCATGCGGGAAGAAAAGAG	
	rev	GCGCCGAAAAGCTAGAAAAG	
iNos qPCR	probe	FAM-CTCTGCCTGCTGTTGCCAACATGC-TAMRA	81 bp
	for	GAGTGGTTTAAGGAGTTGGATCTGA	
	rev	TTCCAGACCTCCCACCTCAA	
SB1UL51-F	for	CTGTACATTCTAGCCAACGACTG	384 bp
SB1UL51-R	rev	GCCCGGATCCCAGTGGCTGATAAC	

## Data Availability

The raw data supporting the conclusions of this article will be made available by the authors on upon request.

## Abbreviations

AA, amino acids; CEFs, chicken embryonic fibroblasts; BoAHV1, bovine alphaherpesvirus 1; CESCs, chicken embryonic skin cells; dpi, day post-infection; FFE, feather follicle epithelium; GaAHV1, gallid alphaherpesvirus 1; GaAHV3, gallid alphaherpesvirus 3; HVT, herpesvirus of turkey; HSV1, human simplex virus 1; MD, Marek’s disease; MDV, Marek’s disease virus; PRV, pseudorabies virus; SPF, specific pathogen free; VZV, varicella-zoster virus.
